# The Prognostic Value of Nodal Staging in Triple-Negative Breast Cancer — A Cohort from China

**DOI:** 10.1038/s41598-018-23999-8

**Published:** 2018-06-13

**Authors:** Liu Yin, Hao Shuang, Chen Sheng, Huang Liang, Xiang-Jie Sun, Wen-Tao Yang, Zhi-Ming Shao

**Affiliations:** 1 Department of Breast Surgery, Fudan University Shanghai Cancer Center/Cancer Institute, Shanghai, P.R. China; 2 Department of Oncology, Shanghai Medical College, Fudan University, Shanghai, P.R. China; 3 Department of Pathology, Fudan University Shanghai Cancer Center/Cancer Institute, Shanghai, P.R. China; 4 Institutes of Biomedical Science, Fudan University, Shanghai, P.R. China

## Abstract

To evaluate the clinical outcomes and relationship between tumor size, lymph node status, and prognosis in a large cohort of patients with triple–negative breast cancer (TNBC).849 Patients were categorized by tumor size and nodal status. The Kaplan-Meier method and Cox proportional hazards models were used to determine the association of nodal status and tumor size with survival outcomes. A Sidak adjustment was used for pairwise group comparisons. We conducted six pairwise comparisons between different node status. In univariate and multivariate analyses, it was indicated that N0 patients had similar prognoses as N1 patients (P = 0.072), and the OS of both of these groups was significantly better than that of N2/N3 patients (N0 vs N2, P < 0.001; N0 vs N3, P < 0.001; N1 vs N2, P = 0.014; N1 vs N3, P = 0.005). In summary, we report that in Chinese patients with triple-negative breast cancer, a greater difference in survival was observed between N1 and N2 than between N0 and N1, warranting the possible need of more intensive chemotherapy for N2-3 patients. We also found that tumor size made an impact on survival when lymph nodes were extensively involved, a finding that needs longer follow-up and further validation.

## Introduction

The incidence rate of breast cancer is increasing almost every year throughout the world, although the mortality rate is declining in many countries, including China. Such advances are mainly due to the systematic use of adjuvant chemotherapy and the development of directed therapies for hormone receptor–positive and C-erbB-2 (HER2)–positive tumors. With a deeper insight into breast cancer over the past two decades, breast cancer is regarded as a heterogeneous disease and can be defined by five intrinsic subtypes: ‘luminal A’, ‘luminal B’, ‘HER2 overexpressing’, ‘basal-like’ and ‘normal breast-like’ tumors. Basal-like breast cancer is a subtype of breast tumor with unique characteristics in terms of clinical-pathological presentation, prognosis, and response to therapy, with poorer prognosis than luminal tumors. However, in clinical work, basal-like breast cancer is not convenient to identify. Three quarters of triple-negative breast cancers, which are defined as ER negative, PR negative and lacking overexpression of HER2, express basal markers; thus, the triple-negative type is frequently taken as a surrogate marker of basal-like breast cancer. This “triple-negative” category comprises 10 to 15% of all breast cancers. In retrospective studies, patients with TNBC have worse clinical outcomes when compared with those with non-TNBC. These tumors tend to present at a younger age, with higher histologic grade, larger size, higher rate of p53 mutations, and positive Ki-67 staining, and they have a tendency toward local and visceral metastases rather than bone metastases^1^.

Currently, the assessment of prognosis and the appropriate treatment is based on patient and standard tumor-related characteristics. Among these factors, tumor size and nodal status are known to be independent prognostic factors and play an important role in treatment decisions. It is well-known that in breast cancer, the size of the primary tumor and the number of positive lymph nodes has an inverse linear relationship with prognosis and survival. Previous studies also indicated that in cases of more than 7 nodes involved, the survival rate decreased, regardless of tumor size^2^. Additionally, lymph node metastasis was more important than tumor size for prognosis.

However, evidence have shown that in patients with TNBC, tumor size and lymph node status may not be linearly correlated with survival outcomes, and even the small tumors in the triple-negative group had a high rate of node positivity^3^. Moreover, once there is evidence of lymph node metastasis, the prognosis may not be affected by the number of positive lymph nodes^1^. However, clinical evidence are still conflicting and confusing. To better illustrate this point, we sought to analyze the clinical outcomes and relationship between lymph node status and prognosis in a large cohort of patients with confirmed TNBC.

## Methods

### Patients and follow-up

From the database of Shanghai Cancer Center/Hospital of Fudan University, we identified consecutive 849 women with TNBC with complete follow-up data who received treatment for early-stage breast cancer between January 2002 and December 2011. According to the inclusion criteria, all cases were confirmed as females with TNBC but no distant metastasis at the initial diagnosis. All patients received a complete physical examination, bilateral mammography, chest radioscopy, ECG and ultrasonography of the breast, axillary fossa, abdomen and pelvis. All patients were treated with a multidisciplinary approach. Most patients(93.6%) received adjuvant chemotherapy using different regimens (taxane-based, or a combined anthracycline/taxane-based and nonanthracycline/taxane-based regimen) according to the standards used at the time of surgery, followed by radiotherapy (if required). Exclusion criteria included ER or PR positivity, HER2 overexpression or amplification, unknown date of surgery, prior administration of neoadjuvant chemotherapy, absence of the date of last follow-up, additional malignancy and male sex. For all patients, information was available for patient age and menopausal status at diagnosis, tumor size, number of lymph nodes removed, number of positive lymph nodes, histological type, histological grade and treatment regimen. Information on date and cause of death came from linking to the Center for Disease Control records, using the unique personal identification number issued to every Chinese citizen. Causes of death were obtained from death certificates and were classified as either death as a result of breast cancer or death from other causes. In this study, we identified OS as the primary end point because the cause of death is two-year lagging behind survival information (simply dead or alive). OS was defined as time from surgery to death. The study was conducted according to the principles expressed in the Declaration of Helsinki and was approved by the institutional review board of Shanghai Cancer Center, Fudan University. All patients enrolled in this study signed the informed consent voluntarily.

### Pathology methods

ER, PR and HER2 status were determined on representative paraffin sections of each tumor using immunohistochemical (IHC) staining that was performed after the patient underwent surgery. ER and PR antibodies were purchased from Dako (clones ER 1D5 1:35 and PR 636 1:50) and were evaluated by an avidin–biotin–peroxidase complex (ABC) assay as described by Shimada *et al*.^4^ ER and PR were considered positive in breast cancer cells if the positive nuclei number was ≥10%. After July 2010, tumors in which ≥1% of tumor cells staining for ER or PgR of any intensity were considered positive^5^. Cytoplasmic staining was ignored^6^. The overexpression of HER2 protein was evaluated using a monoclonal antibody (Dako, Clone PN2A 1:400), and a peroxidase-antiperoxidase (PAP) technique. Positive HER2 was defined as a complete membrane staining in >10% of tumor cells^7^, using a qualitative HercepTest scale of 0–3+, in which scores 0–1 were negative, and 3 was positive^8^. Fluorescence *in situ* hybridization tests were used when the IHC results were ambiguous (i.e., 2+), or for patients who could not be defined as HER2. The pathological and IHC outcomes were diagnosed under an Olympus light microscope with ×10 and ×40 magnifications by two independent senior pathologists in the Department of Pathology of the Cancer Hospital at Fudan University.

### Statistical analysis

Based on the pathology review, the number of positive lymph nodes was categorized into one of four groups: N0 (negative lymph nodes), N1 (one to three positive lymph nodes), N2 (four to nine positive lymph nodes), and N3 (10 or more positive lymph nodes). For BCSS, we used the time from definitive surgery until death as a result of breast cancer or the date of last survival update. Patients who died before experiencing a disease recurrence were considered censored at their dates of death. The Kaplan-Meier method was used to estimate the survival outcomes of all patients by different categories; groups were compared using the log-rank statistic. The Sidak adjustment method was used for multiple group pairwise comparisons. Cox proportional hazards regression models were applied in multivariate analysis. The results are expressed in hazard ratios (HRs) and 95% CIs. P values less than 0.05 were considered statistically significant; all tests were two-sided. Statistical analyses were performed using SPSS Statistics 19.0 (IBM SPSS Statistics 19). P < 0.05 was considered significant.

## Results

### Patient demographics and tumor characteristics

A total of 849 women with TNBC were included in this study. Patient characteristics are listed in Table 1. The median age was 53 years (range, 23 to 87 years). Three hundred eight patients (44.8%) had a tumor size that was less than 2 cm (T1), 438 patients (51.6%) had a tumor size that was between 2 and 5 cm (T2), and 17 patients (2.0%) had a tumor size that was greater than 5 cm (T3/4). Of these patients, 398 (46.9%) were premenopausal, while 451 (53.1%) were not. As to nodal status, 551 patients (64.9%) were node-negative, and 298 (35.1%) were node-positive. A total of 795 patients (93.6%) received chemotherapy, which included taxanes, anthracyclines or a combination. Of the patients, 145 (17.1%) underwent breast-conserving surgery, while the other 704 (82.9%) underwent radical mastectomy or mastectomy.

**Table 1:** Patient Characteristics.

Clinical/pathological Characteristics	Triple Negative BC(n = 849)	
	Number	Proportion%
Age Median (range)	53(23–87)	
≤50 ys	370	43.6
>50 ys	479	56.4
**Menopausal status**		
premenopausal	398	46.9
postmenopausal	451	53.1
**Tumor size**		
T1	380	44.8
T2	438	51.6
T3	17	2
Unknown	14	1.6
**Grade**		
I-II	363	42.8
III	464	54.6
Unknown	22	2.6
**Nodal status**		
Negative	551	64.9
Positive LVI^1^	298	35.1
Negative	663	78.1
Positive	186	21.9
**Chemotherapy**		
Yes	795	93.6
No	38	4.5
Unknown	16	1.9
**Surgery**		
BCS^2^ + RT^3^	145	17.1
Radical Mastectomy	627	73.9
Mastectomy	40	4.7
Other	37	4.3

^1^LVI: lymphvascular invasion.

^2^BCS: breast conserving surgery.

^3^RT: radiotherapy.

### Univariate and multivariate survival analyses of patients

The median follow-up of the entire cohort was 53 months (range, 0.7 month to 317 months). There were 106 deaths, and the 5-year OS rate was 83% (95% CI, 67 to 72%). For node-negative patients, the 5-year OS rate was 89%. As the number of lymph nodes increased, the 5-year OS rate decreased to 81%, 66%, and 58%, respectively, for N1, N2, and N3 patients (P < 0.001) (Table 2). Postmenopausal status (HR = 2.216, 95% CI = 1.445–3.400, P < 0.001), T3/T4 (HR = 5.629, 95% CI = 2.490–12.725, P < 0.001), lymph node involvement (HR = 2.563,95% CI = 1.725–3.809, P < 0.001), lymphatic vascular invasion (HR = 2.104, 95% CI = 1.358–3.258, P = 0.001) and local treatment, which includes mastectomy and radiotherapy (HR = 3.72, 95% CI = 1.085–12.871, P < 0.001), predicted worse overall survival in univariate analysis(Table 3). Multivariate analysis using the Cox model was also performed, indicating that postmenopausal status (HR = 2.537, 95% CI = 1.498–3.708, P < 0.001), T3/T4 (HR = 3.791, 95% CI = 1.533–9.376, P = 0.004) and lymph node involvement (HR = 2.135, 95% CI = 1.386–3.292, P = 0.001) were all independent prognostic factors for OS (Table 4).

**Table 2:** Five-year Survival Estimates by Lymph Node Stage.

Nodal Status	No.of Patients	Overall Survival		
		No.of Events	5-Year Estimates	Overall P
Total	849	106	0.83	<0.001
N0	529	41	0.89	
N1	183	24	0.81	
N2	82	21	0.66	
N3	55	20	0.58

**Table 3:** Univariate Analysis of Overall Survival in TNBC Cancer Patients.

	Overall Survival		
	HR	95% CI	Overall P
**Age**			
≤50 ys	1		
>50 ys	1.677	1.104–2.546	0.013
**Menopausal status**			
premenopausal	1		
postmenopausal	2.216	1.445–3.400	<0.001
Tumor size			<0.001
T1	1		
T2	1.588	1.041–2.422	0.032
T3	5.629	2.490–12.725	<0.001
**Grade**			
I-II	1		
III	1.367	0.913–2.048	0.129
**Lymph node Involvement**			
−	1		
+	2.563	1.725–3.809	<0.001
**LVI** ^**1**^			
−	1		
+	2.104	1.358–3.258	0.001
**Radiotherapy**			
Yes	1		
No	0.784	0.486–1.266	0.319
**Chemotherapy**			
Yes	1		
No	1.561	0.806–3.020	0.186
Local treatment			<0.001
BCS^2^+RT	1		
Mastectomy	2.483	1.150–5.362	0.055
**Mastectomy**			
plus RT^3^	3.72	1.085–12.871	<0.001

^1^LVI: lymphvascular invasion.

^2^BCS: breast conserving surgery.

^3^RT: radiotherapy.

**Table 4:** Cox Proportional Hazards Modals.

	OS		
	HR	95% CI	Adjusted P
Menopausal status pre- vs post- Tumor size	2.357	1.498–3.708	<0.001
			0.009
T1 vs T2	1.557	1.002–2.420	0.049
T1 vs T3	3.791	1.533–9.376	0.004
T2 vs T3	2.434	1.033–5.740	0.042
Lymph node involvement Negative vs positive	2.135	1.386–3.292	0.001
LVI^1^ Negative vs positive	1.061	0.637–1.765	0.821
Surgery			0.145
BCS^2^ + RT^3^ vs mastectomy	1.371	0.615–3.056	0.441
BCS + RT vs mastectomy + RT	2.204	0.884–5.496	0.09

^1^LVI: lymphovascular invasion.

^2^BCS: breast conserving surgery.

^3^RT: radiotherapy.

### Pairwise survival comparison by lymph node involvement

We conducted six pairwise comparisons (N0 vs N1, N2, N3; N1 vs N2, N3; and N2 vs N3) between different node statuses using univariate and multivariate methods. In univariate analysis, it was indicated the outcome of N0 and N1 patients was not significantly different (P = 0.072), while the prognosis of N0 patients was significantly better than the N2/N3 group (both P < 0.001). In a pairwise comparison using the multivariate method, this phenomenon was further validated. The prognosis of N0 patients was similar to that of N1 patients (P = 0.072), and the OS of both these groups was significantly better than that of N2/N3 patients (N0 vs N2, P < 0.001; N0 vs N3, P < 0.001; N1 vs N2, P = 0.014; N1 vs N3, P = 0.005). Moreover, no significant difference in survival was observed between N2 and N3 patients (P = 0.578) (Table 5).

**Table 5:** Pairwise Comparisons between Different Node Status Using Univariate and Multivariate Methods.

Pairwise	Univariate analysis of Overall Survival		Multivariate analysis of Overall Survival	
	P	HR	95%CI	P
N0 v N1	0.072	1.675	0.957–2.931	0.071
N0 v N2	<0.001	3.661	2.063–6.498	<0.001
N0 v N3	<0.001	4.419	2.348–8.318	<0.001
N1 v N2	0.062	2.186	1.171–4.080	0.014
N1 v N3	<0.001	2.638	1.347–5.169	0.005
N2 v N3	0.012	1.207	0.622–2.343	0.578

### Survival and comparisons by tumor size and lymph node status

**Figure 1:**
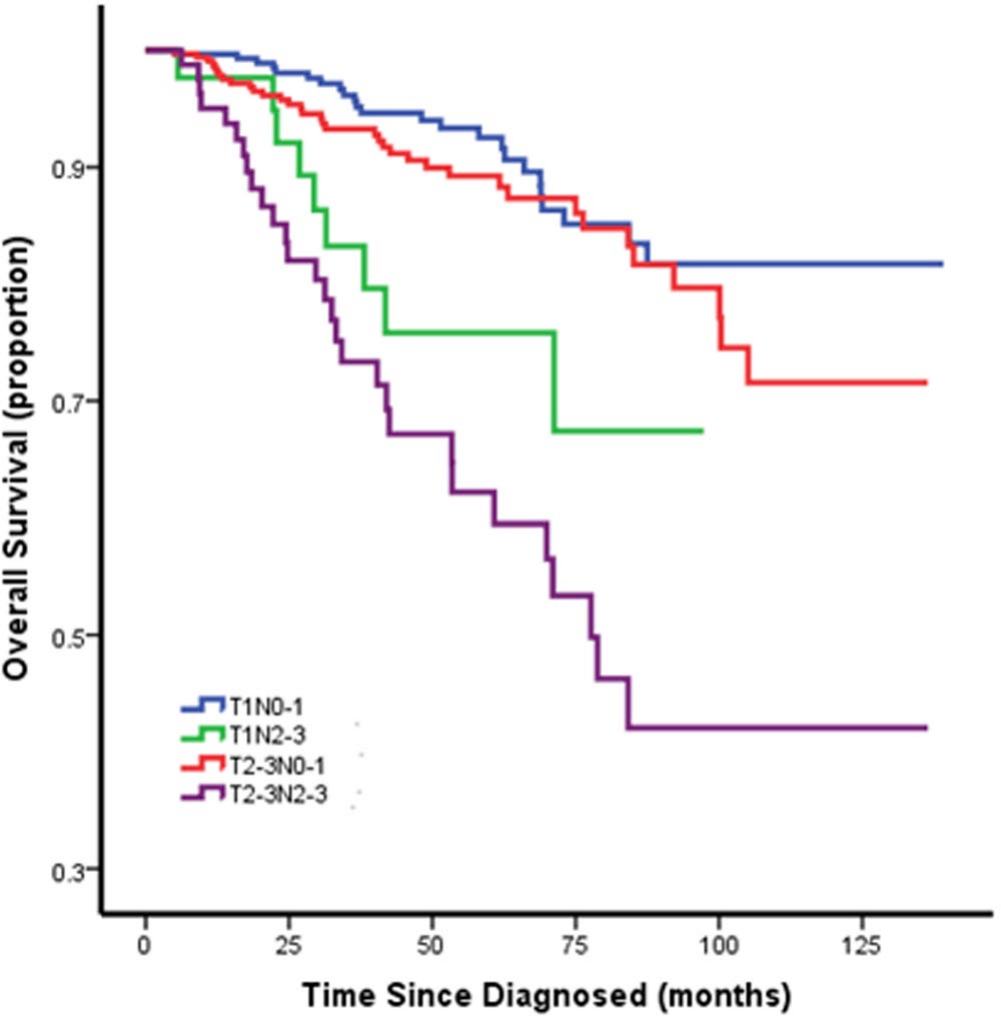
Survival and Comparisons by Tumor Size and Lymph Node Status.

We found that survival differed more between N1 and N2 than between N0 and N1, so we categorized N0 and N1 into one group and N2 and N3 into the other. Then, we conducted survival comparisons (T1N0-1 vs T1N2-3 vs T2-3N0-1 vs T2-3N2-3) between different node status and tumor sizes using univariate methods. The Kaplan-Meier curve showed there was no survival difference between T1N0-1 vs T2-3N0-1, which meant that tumor size did not affect survival when there was less lymph node involvement. The prognosis of the T1N2-3 group was worse than that of the T1N0-1 and T2-3N0-1 groups, but it was better than that of the T2-3N2-3 group (P < 0.001), which meant that tumor size did impact survival when more lymph nodes were involved (Fig. 1). This finding was somewhat different from our previous cognition.

## Discussion

In this study, we evaluated the clinical outcomes and relationships between tumor size, lymph node status, and prognosis in a large cohort of Chinese patients with confirmed triple-negative breast cancer. In our study cohort, the OS rate decreased as the number of positive lymph nodes increased. This finding has been reported in previous studies^9^ many times and is in agreement with our previous cognition.

However, when we conducted pairwise comparisons using the Sidak adjustment method, our study makes the important observation that the outcome of N0 and N1 patients was not significantly different (P = 0.072), while the prognosis of N0 patients was significantly better than that of the N2/N3 group (P < 0.001, both). In other words, survival differed more between N1 and N2 than between N0 and N1 in patients with triple-negative breast cancer. This finding was consistent with the AJCC staging system^10^, which categorizes N2 and N3 together into Stage III while leaving N1 in Stage II. However, it was discordant with a previous study published in the *Journal of Clinical Oncology* by Leonel F. Hernandez-Aya, whose study concluded that once there is evidence of lymph node metastasis, the prognosis may not be affected by the number of positive lymph nodes^1^. The difference may due to different genetic backgrounds of patients and to the heterogeneity of triple-negative breast cancer itself. For example, black women were more likely to be diagnosed with triple-negative breast cancer than Chinese women and non-Hispanic white women^11^. Moreover, the actuarial probability of a woman dying due to small-sized breast cancer tumors was significantly higher for black women compared with non-Hispanic white women and Chinese women^12^. Therefore, the genetic background of triple-negative breast cancer patients was largely different between the study by Hernandez-Aya and ours. Moreover, the cases in that study were collected from 1980, when the chemotherapy regimen and lymph node dissection standard was quite different from the standards used today.

We found that survival differed more largely between N1 and N2 than between N0 and N1, so we categorized N0 and N1 into one group and N2 and N3 into the other. Then, we conducted survival comparisons (T1N0-1 vs T1N2-3 vs T2-3N0-1 vs T2-3N2-3) between different node statuses and tumor sizes using a univariate method. The prognosis of T1N2-3 group was worse than that of T1N0-1 and T2-3N0-1 but better than that of T2-3N2-3 (P < 0.001), which meant tumor size made an impact on survival when lymph nodes were more involved. This finding was not in concordance with the AJCC staging system. In the AJCC staging system, N2 and N3 were categorized as stage III no matter the tumor size (T4 excluded here). This phenomenon may be explained in two aspects. Although it is found that the “size-nodes” relationship in triple-negative breast cancer is distinct and that tumor size is not a strong indicator of prognosis in basal-like breast cancer^3,13^, the reason for this result may be due to the short follow-up time. In a follow-up to the triple-negative study in Toronto, women with basal-like breast cancer <2 cm in diameter had a survival advantage compared with women with larger basal-like breast cancers (hazard ratio = 0.5, 95% CI 0.4–0.7; P = 0.002) for the first 5 years, but after 8 years of follow-up, the proportion of cancer-related deaths was approximately 40% in both groups^14^. Thus, tumor size was predictive of short-term survival for women with triple-negative breast cancer, but it was not a predictor of long-term survival. The underlying mechanism might also be explained as follows: it has been proposed that a higher fixed proportion of cancer cells with stem-cell properties of self-renewing and pluripotency, which we call cancer stem cells, may be present^15,16^, and these cells may confer the capacity to small tumors to metastasize to distant sites. However, there are two dimensions to examine the degree of malignancy of cancer stem cells. One is migration ability, and the other is proliferation ability. Tumors with extensive lymph node involvement when they are small tend to have strong migration ability but not proliferation ability, while large tumors with extensive lymph node involvement tend to have strong migration ability and proliferation ability. Thus, for small tumors with extensive lymph node involvement, even if the cancer cells migrate to distant organs, it may take a longer time to form a clinically detectable tumor lesion, leading to comparatively later recurrence and death.

To the best of our knowledge, this is the largest study evaluating the prognostic significance of tumor size and lymph node status in Chinese patients with TNBC. Our survival data was from the Center of Disease Control records and death records—data with high accuracy. However, there are still limitations that should be mentioned. First, this is a retrospective study with inevitable bias. Additionally, we need to account for the significant exclusion of patients treated with neoadjuvant chemotherapy. This exclusion resulted in a highly select patient population that may not accurately reflect the entire cohort of patients with triple-negative breast cancers.

In summary, we report that in this large cohort of Chinese patients with triple-negative breast cancer, survival differed more between N1 and N2 than between N0 and N1, rendering the possible need of more intensive chemotherapy for N2-3 patients. We also found that tumor size had an impact on survival when lymph nodes were extensively involved, a finding which needs longer follow-up and further validation. This finding may enhance our understanding of the recurrence pattern in specific triple-negative breast cancer subpopulations.
